# Documenting Grief and Bereavement Experiences among Older Chinese Immigrants in LA: A Photovoice Pilot Study

**DOI:** 10.1093/geroni/igaf122.3451

**Published:** 2025-12-31

**Authors:** Qianyun Wang, Cindy Sangalang

**Affiliations:** University of California, Los Angeles, Los Angeles, California, United States; University of California, Los Angeles, Los Angeles, California, United States

## Abstract

This pilot study explored grief and bereavement experiences among older Chinese immigrants in Los Angeles using a Photovoice approach. The COVID-19 pandemic not only triggered global waves of grief but also intensified anti-Asian racism, disproportionately affecting older Asian immigrants with severe symptoms of depression and anxiety. Older Chinese immigrants faced unique challenges in grief adjustment due to cultural and linguistic barriers, limited social support, and discrimination in healthcare settings. This study employed Photovoice, a participatory action research strategy, to authentically capture and discuss these experiences through visual data. The research, conducted in collaboration with the Chinatown Service Center, addressed the following research questions: 1) What were the lived experiences of grief and bereavement among older Chinese immigrants? 2) How did intersecting factors like age, immigration status, and ethnicity impact their experiences? 3) What challenges and support did they encounter in adapting to grief? A purposive sample of 20-30 participants in total aged 65 or older, born outside the United States, and having lost a loved one over six months ago will be recruited. The methodology included two individual interviews and a focus group discussion, where participants took and discussed photographs representing their grief experiences. Preliminary results were documented and will be presented in a community photographic exhibition. This study aimed to provide insights into the nuanced grief experiences of older Chinese immigrants, contributing to culturally sensitive support frameworks.

